# A Multilayer Naïve Bayes Model for Analyzing User's Retweeting Sentiment Tendency

**DOI:** 10.1155/2015/510281

**Published:** 2015-08-31

**Authors:** Mengmeng Wang, Wanli Zuo, Ying Wang

**Affiliations:** ^1^College of Computer Science and Technology, Jilin University, Changchun 130012, China; ^2^Key Laboratory of Symbolic Computation and Knowledge Engineering, Jilin University, Ministry of Education, Changchun 130012, China; ^3^College of Mathematics, Jilin University, Changchun 130012, China

## Abstract

Today microblogging has increasingly become a means of information diffusion via user's retweeting behavior. Since retweeting content, as context information of microblogging, is an understanding of microblogging, hence, user's retweeting sentiment tendency analysis has gradually become a hot research topic. Targeted at online microblogging, a dynamic social network, we investigate how to exploit dynamic retweeting sentiment features in retweeting sentiment tendency analysis. On the basis of time series of user's network structure information and published text information, we first model dynamic retweeting sentiment features. Then we build Naïve Bayes models from profile-, relationship-, and emotion-based dimensions, respectively. Finally, we build a multilayer Naïve Bayes model based on multidimensional Naïve Bayes models to analyze user's retweeting sentiment tendency towards a microblog. Experiments on real-world dataset demonstrate the effectiveness of the proposed framework. Further experiments are conducted to understand the importance of dynamic retweeting sentiment features and temporal information in retweeting sentiment tendency analysis. What is more, we provide a new train of thought for retweeting sentiment tendency analysis in dynamic social networks.

## 1. Introduction

With the rapid growth of user-generated data on the Web, people usually use microblogging which has become a novel social media [1] for expressing their opinion. Therefore, a necessity of analyzing and understanding these online generated data/opinions has arisen [2]. Mining emotional information in users' contents may contribute to analyzing the relationship between social economy change and emotion change expressed by the public [3], measuring strength of public happiness [4], detecting current trend of stock market [5], predicting results of presidential election [6], and modeling for opinion mining [7]. Hence, analyzing sentiment tendency of microblogging has gradually become a hot research topic.

Furthermore, the emergence of microblogging has broken the mode of transmission: once a user posts a microblog, other users can retweet it and add more contents via 140 words text box which makes further development and enrichment of information during forwarding process. Thanks to resonance of information, retweeting content, as context information of microblogging, contains users' views and emotions to express approval or opposition towards a certain microblog. Besides, exploring on retweeting sentiment tendency can make enterprises and governments better understand users' opinions on products, stocks, current hot issues, hit movies, hate to someone, and so forth. As stated, retweeting sentiment tendency analysis is of great significance for public opinion monitoring. Our work on predicting user's retweeting sentiment tendency is motivated by its broad application prospect.

However, previous sentiment tendency analysis methods [8–11], which only focused on emotion of contents rather than users' individual emotion, attributes, social correlations, and dynamic nature of network, cannot make a comprehensive analysis on user's retweeting sentiment tendency. Hence, in this paper, we propose a multilayer Naïve Bayes model for analyzing user's retweeting sentiment tendency towards a microblog (denoted as MLNBRST), and our main contributions are summarized next.

1. Take user's recent individual emotion as well as emotion difference between user and microblog as metrics to further analyze user's retweeting sentiment tendency.
2. Improve traditional Salton metrics according to directivity of link for being applied to directed network better.
3. Blend temporal information in user's retweeting sentiment features on the basis of time series of user's contents and network topological information so as to capture dynamic evolution process of information and network structure.
4. Build a multilayer Naïve Bayes model on account of Naïve Bayes models from different dimensions to complete user's retweeting sentiment tendency analysis in a more fine-grained perspective.
5. Evaluate MLNBRST on real-world Sina microblogging dataset and elaborate the importance of different retweeting sentiment features and temporal information on user's retweeting sentiment tendency analysis.

The rest of the paper is organized as follows: Section 2 describes the related work; dynamic retweeting sentiment features are depicted in Section 3; Section 4 defines the method we propose; details of the experimental results and dataset which is used in this study are given in Section 5. Finally conclusion appears in Section 6.

## 2. Related Work

In recent years, with the popularization of microblogging, sentiment analysis of microblogging has become one of the hot research topics [12]. Existing microblogging sentiment analysis algorithms can be roughly categorized into two groups: emotional dictionary-based methods and machine learning methods.

In emotional dictionary-based methods, through summing up all emotion words' sentiment polarity, a microblog's sentiment polarity is calculated. Golder and Macy [8] adopted a prominent lexicon, Linguistic Inquiry and Word Count (LIWC), to analyze sentiment automatically with tweets which were published by millions of different regions and different cultural background microbloggers, and the results revealed that the proposed method can clearly identify user's emotion pattern in a period of time. Ghiassi et al. [13] introduced an approach to supervised feature reduction using n-grams and statistical analysis to develop a Twitter-specific lexicon for sentiment analysis which yielded improvement of sentiment classification accuracy. Montejo-Ráez et al. [14, 15] explored an unsupervised domain-independent method for polarity classification in Twitter via combining SentiWordNet scores with WordNet. Through weighting SentiWordNet values with the score of a random walk analysis (PageRank) on the concepts found in texts over WordNet graph, polarity classification problem was solved. The results obtained showed that both disambiguation and expansion were good strategies for improving performance. Stieglitz and Dang-Xuan [9] leveraged the tool “SentiStrength” which used a human-designed lexicon of emotional terms with a set of additional linguistic rules for negations, booster words, amplifications, emoticons, spelling corrections, and other factors such as word weighting to analyze the level of sentiments in politically relevant tweets.

Although emotional dictionary-based method can better represent unstructured characteristics of text, it is too dependent on emotion dictionary and ignores the effects of new words. Machine learning method can overcome the influence that unknown words have on the performance of sentiment analysis. It first selects words and phrases as features to form vector space model and then converts sentiment analysis problem into a classification problem by treating different emotional tendencies as different categories. Davidov et al. [10] treated related tags and emoticons in tweets as labels and designed a supervised *k*-nearest neighbor classifier to complete emotion classification on Twitter which did not need too much manual annotation. Based on 95 most frequently used emoticons which were mapped to four emotional categories: happy, hate, low, and anger, Zhao et al. [11] employed the emoticons for the generation of sentiment labels for tweets and built an incremental learning Naïve Bayes classifier for the categorization of four types of sentiments with an empirical precision of 64.3%. Wang et al. [16] analyzed emotion tendentiousness of related microblogs in the 2012 U.S. presidential election. They found that, in terms of short text, effect of Naïve Bayes was better than that of SVM on emotion tendentiousness classification. So they presented an improved SVM classifier NBSVM which was better and more stable than SVM and Naïve Bayes. Korenek and Šimko [17] first created an appraisal dictionary by utilizing a psychological theory called appraisal theory which allowed a deeper and fine-grained analysis of microbloggings, followed by classifying posts using SVM classifier which revealed that the proposed method was feasible even for specific content presented on microbloggings. On the basis of common social network characteristics and other carefully generalized linguistic patterns, Li and Xu [18] proposed and implemented a novel method for identifying sentiment in microblogging posts. First, after thorough analysis on sample data, an automatic rule-based system was constructed to detect and extract the cause event of each emotional post. And then an emotional corpus was built with Chinese microblogging posts labeled by human annotators. Finally, a classifier, namely, SVR (support vector regression), was trained to classify emotions in microblogging posts based on extracted cause events. The overall performance of proposed system was very promising. Focusing on emotion dynamics in OSNs, Xiong et al. [19] proposed an emotion classifier based on Bayes theory, and some effective strategies (Shannon entropy and the salience degree of each word) were introduced to improve the performance of classifier, with which proposed method can classify any Chinese tweet into a particular emotion with a satisfactory accuracy. In order to achieve target-dependent Twitter sentiment classification, Dong et al. [20] proposed Adaptive Recursive Neural Network (AdaRNN) through employing more than one composition function. For a given tweet, its dependency tree for interested target was first converted. Next, AdaRNN learned how to adaptively propagate sentiments of words to target node based on context and linguistic tags. The experimental studies illustrated that AdaRNN improved the baseline methods.

To sum up, retweeting sentiment tendency analyzing in dynamic social networks is in the stage of development; how to depict directivity of relationship, how to fuse multidimensional features reasonably, and how to build model that could adapt to dynamic evolution process of network can be very challenging jobs. To this end, we present a multilayer Naïve Bayes model for analyzing user's retweeting sentiment tendency which is appropriate for dynamic and directed social networks.

## 3. Modeling Retweeting Sentiment Tendency Features

Since it cannot make a comprehensive analysis on user's retweeting sentiment tendency based on specific types of features only, consequently, in this paper, we synthesize profile-, relationship-, and emotion-based features so as to achieve higher accuracy in retweeting sentiment tendency analysis.

### 3.1. Profile-Based Features

In this paper, according to dataset in [21], we name the number of bifollowers, the number of followers, the number of followees, the number of contents that user posts, the user's province, the user's city, the user's gender, the created time of user's account, and the verified type of user's account which are provided in the original dataset as profile-based features.

### 3.2. Relationship-Based Features

Tan et al. [22] pointed out that users who had close relationship may have similar emotional point of view, so relationship between users can be used to extract users' emotions. In this paper, we infer relationship-based features via user's network topological structure and interaction frequency.

#### 3.2.1. Dynamic Salton Metrics

Common Neighbors metrics assumed that similarity between users was proportional to the number of their common neighbors [23]. Salton metrics introduces users' degree compared to Common Neighbors metrics to measure user's network topological structure. Since nonreciprocal friendships, which may reflect moderately valued friendship ties [24], are more important than reciprocal friends, hence, we make an improvement on traditional Salton metrics according to directivity of link. Besides, understanding dynamic structure of online social networks plays an important role in the development of retweeting sentiment tendency analyzing algorithms. Thus, given the dynamic nature of social network, we consider network as a dynamic flow of time slices (one time slice stands for one day and the older the time slice is, the lower is its importance, so does its weight), and dynamic Salton metrics between user *u* and user *v* on the *i*th time slice *t*_*i*_ is defined as follows:

(1) $$\begin{array}{c} \text{Sa}\left(u,v,t_{i}\right)=\frac{\left|\Gamma^{\text{in}}\left(u,t_{i}\right)\cap \Gamma^{\text{in}}\left(v,t_{i}\right)\right|/\sqrt{\left|\Gamma^{\text{in}}\left(u,t_{i}\right)\right|\left|\Gamma^{\text{in}}\left(v,t_{i}\right)\right|}}{\left|\Gamma^{\text{out}}\left(u,t_{i}\right)\cap \Gamma^{\text{out}}\left(v,t_{i}\right)\right|/\sqrt{\left|\Gamma^{\text{out}}\left(u,t_{i}\right)\right|\left|\Gamma^{\text{out}}\left(v,t_{i}\right)\right|}}, \end{array}$$

where Γ^in^(*u*, *t*_*i*_) and Γ^in^(*v*, *t*_*i*_) stand for in-link users set of user *u* and user *v* on *t*_*i*_, respectively; Γ^out^(*u*, *t*_*i*_) and Γ^out^(*v*, *t*_*i*_) stand for out-link users set of user *u* and user *v* on *t*_*i*_, respectively, where in-link and out-link are defined by follower relationship; and |Γ(*x*)| stands for the number of elements in set Γ(*x*). Thus, dynamic Salton metrics between user *u* and user *v* on the flow of time slices [0, *t*_*n*_] is calculated as

(2) $$\begin{array}{c} {\text{Sa}}^{\left[0,t_{n}\right]}\left(u,v\right)=\overset{n}{\underset{i=0}{\sum }}\alpha^{n-i}\times \text{Sa}\left(u,v,t_{i}\right), \end{array}$$

where *α* ∈ [0,1], *α* ^*n*−*i*^ represents weight of *t*_*i*_, and *n* represents the number of time slices.

#### 3.2.2. Dynamic Interaction Frequency

Similar to dynamic Salton metrics, we employ *α* ^*n*−*i*^ which is defined in Section 3.2.1 to stand for the *i*th time slice *t*_*i*_'s weight, and dynamic interaction frequency between user *u* and user *v* on *t*_*i*_ is defined as follows:

(3) $$\begin{array}{c} \text{Fre}\left(u,v,t_{i}\right)=\frac{c\left(u,v,t_{i}\right)}{p\left(u,t_{i}\right)+p\left(v,t_{i}\right)}, \end{array}$$

where *p*(*u*, *t*_*i*_) and *p*(*v*, *t*_*i*_) stand for the number of posts of user *u* and user *v* on *t*_*i*_, respectively, and *c*(*u*, *v*, *t*_*i*_) stands for the number of interactions between user *u* and user *v* on *t*_*i*_, where an interaction is defined as user *u* retweeting a microblog of user *v* or user *v* retweeting a microblog of user *u*. Thus, dynamic interaction frequency between user *u* and user *v* on the flow of time slices [0, *t*_*n*_] is calculated as

(4) $$\begin{array}{c} {\text{Fre}}^{\left[0,t_{n}\right]}\left(u,v\right)=\overset{n}{\underset{i=0}{\sum }}\alpha^{n-i}\times \text{Fre}\left(u,v,t_{i}\right). \end{array}$$

### 3.3. Emotion-Based Features

Since people tend to post microblogs which express their experiences or views in order to realize desire of self-expression, consequently, there will be quite a lot of emotional words or expressions in their contents. Thus, along with the number of emotional words, we employ recent mood statistics and emotion divergence as emotion-based features.

#### 3.3.1. The Number of Emotional Words

In this paper, we count up the number of positive and negative emotional words in user's retweeting contents with corpus of HowNet Knowledge (http://www.keenage.com/download/sentiment.rar). HowNet Knowledge, which includes 8945 words and phrases, consists of six files: positive emotional words list file, negative emotional words list file, positive review words list file, negative review words list file, degree words list file, and propositional words list file.

#### 3.3.2. Recent Mood Statistics

To enhance the performance of retweeting sentiment analysis, we further conduct analysis on user's contents according to the number of positive and negative emotional words. Similar to dynamic Salton metrics, we employ *α* ^*n*−*i*^ which is defined in Section 3.2.1 to stand for the *i*th time slice *t*_*i*_'s weight, and user *u*'s mood statistics on *t*_*i*_ is calculated as follows:

(5) $$\begin{array}{c} \text{Ue}\left(u,t_{i}\right)=\frac{\text{Upn}\left(u,t_{i}\right)}{\text{Upn}\left(u,t_{i}\right)+\text{Unn}\left(u,t_{i}\right)}, \end{array}$$

where Upn(*u*, *t*_*i*_) and Unn(*u*, *t*_*i*_) represent the number of positive emotional words and the number of negative emotional words user *u* used on *t*_*i*_ which are included in HowNet Knowledge. Thus, user *u*'s recent mood statistics on the flow of time slices [0, *t*_*n*_] is calculated with

(6) $$\begin{array}{c} {\text{Ue}}^{\left[0,t_{n}\right]}\left(u\right)=\overset{n}{\underset{i=0}{\sum }}\alpha^{n-i}\times \text{Ue}\left(u,t_{i}\right). \end{array}$$

#### 3.3.3. Emotion Divergence

In this paper, we quantitatively define emotion divergence between user *u* and microblog *b* as follows:

(7) $$\begin{array}{c} \text{Em}\left(u,b,t_{n}\right)={\text{Ue}}^{\left[0,t_{n}\right]}\left(u\right)-\text{Ie}\left(b\right), \end{array}$$

where Ue^[0, *t*_*n*_]^(*u*) represents user *u*'s latent mood statistics on the flow of time slices [0, *t*_*n*_] and Ie(*b*) represents emotion statistics expressed in microblog *b* which can be calculated as

(8) $$\begin{array}{c} \text{Ie}\left(b\right)=\frac{\text{Ipn}\left(b\right)}{\text{Ipn}\left(b\right)+\text{Inn}\left(b\right)}, \end{array}$$

where Ipn(*b*) and Inn(*b*) denote the number of positive emotional words and the number of negative emotional words used in microblog *b* which are included in HowNet Knowledge.

## 4. Multilayer Naïve Bayes Model for Analyzing User's Retweeting Sentiment Tendency

Some researchers found that Naïve Bayes was more suitable for sentiment classification on microblogging [25]. On the basis of Bayes' theorem, Naïve Bayes model presents uncertainty with probability and realizes process of learning and reasoning via probability. Hence, in this paper, we put forward a multilayer Naïve Bayes model which is a tweak of Naïve Bayes model to analyze user's retweeting sentiment tendency in a fine granularity.

**Figure 1:**
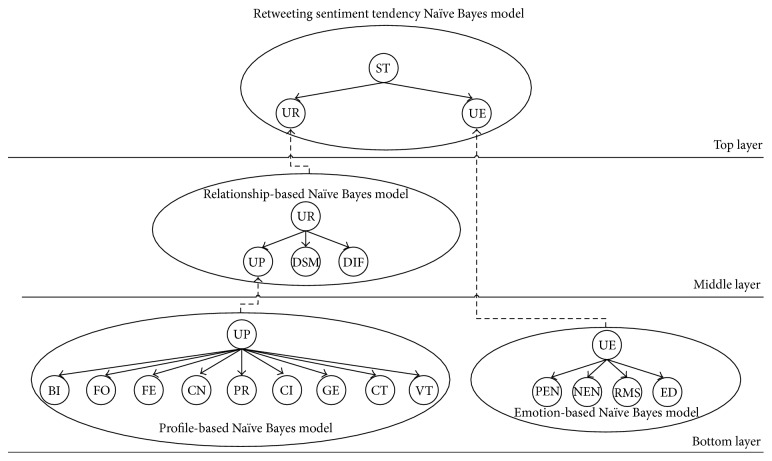
The framework of MLNBRST.

We formally define retweeting sentiment tendency analysis as follows: given a group of retweets with related discrete feature vector and corresponding retweeting sentiment tendency label, we aim to leverage prior knowledge to automatically assign retweeting sentiment tendency labels to unknown retweets. And our proposed method consists of three modules: (1) Naïve Bayes models in bottom layer; (2) Naïve Bayes model in middle layer; (3) Naïve Bayes model in top layer. The process outlined is shown in Figure 1, where the bottom layer is Naïve Bayes model for predicting user's profile and emotion, the middle layer is Naïve Bayes model for predicting user's relationship, and, finally, in the top layer, through multilayer nested Naïve Bayes model, user's retweeting sentiment tendency is predicted. The detailed descriptions are shown as follows.

### 4.1. Naïve Bayes Models in Bottom Layer

#### 4.1.1. Profile-Based Naïve Bayes Model

Naïve Bayes model, which is based on Bayes' theorem, reduces computational overhead via conditional independent assumption to classify unknown samples according to their maximum a posteriori probability. Since the calculation of user's profile (denoted as UP) conforms to Naïve Bayes model which is determined by the number of bifollowers (denoted as BI), the number of followers (denoted as FO), the number of followees (denoted as FE), the number of contents that user posts (denoted as CN), the user's province (denoted as PR), the user's city (denoted as CI), the user's gender (denoted as GE), the created time of user's account (denoted as CT), and the verified type of user's account (denoted as VT), UP can be seen as root node of Naïve Bayes model; BI, FO, FE, CN, PR, CI, GE, CT, and VT can be seen as leaf nodes of Naïve Bayes model.

Given that BI, FO, FE, CN, and CT are continuous attributes, in order to calculate the conditional probability of them, we discretize them by using discrete intervals to represent them before modeling UP: according to the above features' values, BI is mapped to three levels (namely, few, medium, and many); FO is mapped to three levels (namely, few, medium, and many); FE is mapped to three levels (namely, few, medium, and many); CN is mapped to three levels (namely, few, medium, and many); CT is mapped to three levels (namely, short, medium, and long). Consequently, the probability that user's profile equals a certain discrete value is calculated as follows:

(9) $$\begin{array}{c} P\left(\text{UP}=p\;|\;\text{BI},\text{FO},\text{FE},\text{CN},\text{PR},\text{CI},G,\text{CT},\text{VT}\right)=\frac{P\left(\text{UP}=p,\text{BI},\text{FO},\text{FE},\text{CN},\text{PR},\text{CI},G,\text{CT},\text{VT}\right)}{P\left(\text{BI}\right)P\left(\text{FO}\right)P\left(\text{FE}\right)P\left(\text{CN}\right)P\left(\text{PR}\right)P\left(\text{CI}\right)P\left(G\right)P\left(\text{CT}\right)P\left(\text{VT}\right)}=\frac{P\left(\text{UP}=p\right)P\left(\text{BI}\;|\;\text{UP}=p\right)P\left(\text{FO}\;|\;\text{UP}=p\right)P\left(\text{FE}\;|\;\text{UP}=p\right)P\left(\text{CN}\;|\;\text{UP}=p\right)P\left(\text{PR}\;|\;\text{UP}=p\right)P\left(\text{CI}\;|\;\text{UP}=p\right)P\left(G\;|\;\text{UP}=p\right)P\left(\text{CT}\;|\;\text{UP}=p\right)P\left(\text{VT}\;|\;\text{UP}=p\right)}{P\left(\text{BI}\right)P\left(\text{FO}\right)P\left(\text{FE}\right)P\left(\text{CN}\right)P\left(\text{PR}\right)P\left(\text{CI}\right)P\left(G\right)P\left(\text{CT}\right)P\left(\text{VT}\right)}, \end{array}$$

where *p* ∈ {bad, medium, good} denotes a certain discrete value of UP; BI, FO, FE, CN, PR, CI, *G*, CT, and VT denote their corresponding discrete values, respectively; *P*(UP = *p*, BI, FO, FE, CN, PR, CI, *G*, CT, VT) denotes the probability that UP = *p* and BI, FO, FE, CN, PR, CI, *G*, CT, and VT are equal to corresponding discrete values; *P*(·) denotes the probability that a feature equals a certain discrete value; and *P*(·∣UP = *p*) denotes the probability that a feature equals a certain discrete value when UP = *p*. Then, user's profile can be classified into three levels (namely, bad, medium, and good) according to its maximum a posteriori probability which is calculated with (9).

#### 4.1.2. Emotion-Based Naïve Bayes Model

In the same way, the calculation of user's emotion (denoted as UE) also tallies with Naïve Bayes model which is determined by the number of positive emotional words (denoted as PEN), the number of negative emotional words (denoted as NEN), recent mood statistics (denoted as RMS), and emotion divergence (denoted as ED); as a consequence, UE can be viewed as root node of Naïve Bayes model; PEN, NEN, RMS, and ED can be viewed as leaf nodes of Naïve Bayes model.

Before modeling UE, given that PEN, NEN, RMS, and ED are continuous attributes; in order to calculate conditional probability of them, we discretize them by using discrete intervals to represent them: PEN is mapped to three levels (namely, few, medium, and many); NEN is mapped to three levels (namely, few, medium, and many); RMS is mapped to three levels (namely, low, medium, and high); and ED is mapped to three levels (namely, small, medium, and large). And the probability that user's emotion is equal to a certain discrete value is calculated as below:

(10) $$\begin{array}{c} P\left(\text{UE}=e\;|\;\text{PEN},\text{NEN},\text{RMS},\text{ED}\right)=\frac{P\left(\text{UE}=e,\text{PEN},\text{NEN},\text{RMS},\text{ED}\right)}{P\left(\text{PEN}\right)P\left(\text{NEN}\right)P\left(\text{RMS}\right)P\left(\text{ED}\right)}=\frac{P\left(\text{UE}=e\right)P\left(\text{PEN}\;|\;\text{UE}=e\right)P\left(\text{NEN}\;|\;\text{UE}=e\right)P\left(\text{RMS}\;|\;\text{UE}=e\right)P\left(\text{ED}\;|\;\text{UE}=e\right)}{P\left(\text{PEN}\right)P\left(\text{NEN}\right)P\left(\text{RMS}\right)P\left(\text{ED}\right)}, \end{array}$$

where *e* ∈ {low, medium, high} denotes a certain discrete value of UE; PEN, NEN, RMS, and ED denote their corresponding discrete values, respectively; *P*(UE = *e*, PEN, NEN, RMS, ED) denotes the probability that UE = *e* and PEN, NEN, RMS, and ED are equal to corresponding discrete values; and *P*(·∣UE = *e*) denotes the probability that a feature equals a certain discrete value when UE = *e*. Then, we classify user's emotion into three levels (namely, low, medium, and high) according to its maximum a posteriori probability which is calculated with (10).

### 4.2. Naïve Bayes Model in Middle Layer

Similarly, the calculation of user's relationship (denoted as UR) is in accordance with Naïve Bayes model as well which is determined by user's profile (UP), dynamic Salton metrics (denoted as DSM), and dynamic interaction frequency (denoted as DIF); hence, UR can be treated as root node of Naïve Bayes model; UP, DSM, and DIF can be treated as leaf nodes of Naïve Bayes model.

Since DSM and DIF are continuous attributes, in order to calculate the conditional probability of them, we discretize them by using discrete intervals to represent them before modeling UR: DSM is mapped to three levels (namely, low, medium, and high); DIF is mapped to three levels (namely, low, medium, and high). So the probability that user's relationship is equal to a certain discrete value is calculated with

(11) $$\begin{array}{c} P\left(\text{UR}=r\;|\;\text{UP},\text{DSM},\text{DIF}\right)=\frac{P\left(\text{UR}=r,\text{UP},\text{DSM},\text{DIF}\right)}{P\left(\text{UP}\right)P\left(\text{DSM}\right)P\left(\text{DIF}\right)}=\frac{P\left(\text{UR}=r\right)P\left(\text{UP}\;|\;\text{UR}=r\right)P\left(\text{DSM}\;|\;\text{UR}=r\right)P\left(\text{DIF}\;|\;\text{UR}=r\right)}{P\left(\text{UP}\right)P\left(\text{DSM}\right)P\left(\text{DIF}\right)}, \end{array}$$

where *r* ∈ {low, medium, high} denotes a certain discrete value of UR; UP, DSM, and DIF denote their corresponding discrete values, respectively; *P*(UR = *r*, UP, DSM, DIF) denotes the probability that UR = *r* and UP, DSM, and DIF are equal to corresponding discrete values; and *P*(·∣UR = *r*) denotes the probability that a feature equals a certain discrete value when UR = *r*. Then, UR can be classified into three levels (namely, low, medium, and high) according to its maximum a posteriori probability which is calculated with (11).

### 4.3. Naïve Bayes Model in Top Layer

Finally, since the calculation of user's retweeting sentiment tendency (denoted as ST), user's profile (UP), relationship (UR), and emotion (UE) all conforms to Naïve Bayes model, so we adopt a multilayer Naïve Bayes model to analyze user's retweeting sentiment tendency. In this paper, determined by user's relationship and emotion, user's retweeting sentiment tendency can be regarded as root node of Naïve Bayes model; user's relationship and emotion can be regarded as leaf nodes of Naïve Bayes model. Thus, user's retweeting sentiment tendency is calculated as follows:

(12) $$\begin{array}{c} P\left(\text{ST}=\text{st}\;|\;\text{UP},\text{UR},\text{UE}\right)=\frac{P\left(\text{ST}=\text{st},\text{UP},\text{UR},\text{UE}\right)}{P\left(\text{UP}\right)P\left(\text{UR}\right)P\left(\text{UE}\right)}=\frac{P\left(\text{ST}=\text{st}\right)P\left(\text{UP}\;|\;\text{ST}=\text{st}\right)P\left(\text{UR}\;|\;\text{ST}=\text{st}\right)P\left(\text{UE}\;|\;\text{ST}=\text{st}\right)}{P\left(\text{UP}\right)P\left(\text{UR}\right)P\left(\text{UE}\right)}, \end{array}$$

where st ∈ {positive, negative, neutral} denotes a certain discrete value of ST; UP, UR, and UE denote their corresponding discrete values, respectively; *P*(ST = st, UP, UR, UE) denotes the probability that ST = st and UP, UR, and UE are equal to corresponding discrete values; and *P*(·∣ST = st) denotes the probability that a feature equals a certain discrete value when ST = st. Thus, ST could be classified into three particular emotion statuses (namely, positive, negative, and neutral) according to its maximum a posteriori probability which is calculated with (12).

## 5. Experimental Evaluation

In this section, we conduct experiments to assess the effectiveness of the proposed framework MLNBRST. Through the experiments, we aim to answer the following two questions:

- How effective is the proposed framework, MLNBRST, compared with other methods of retweeting sentiment tendency analyzing?
- What are the effects of different features and temporal information on the performance of retweeting sentiment tendency analyzing?

### 5.1. Dataset and Experimental Settings

To study the problem of retweeting sentiment tendency analyzing, we leverage a Sina microblogging dataset [21] which contains time series of users' tweets, retweets, and followings' number from September 28, 2012, to October 29, 2012, to evaluate the validity of the proposed method. Moreover, since not all users post opinions when retweeting, we manually label a subset of Sina microblogging which contains retweeting contents with sentiment polarity. Statistics of the dataset are shown in Table 1.

The experimental settings of retweeting sentiment tendency analyzing are described as follows: we randomly divide the dataset into two parts *A* and *B*. *A* possesses 90% of retweets used for training. The left 10% of retweets denoted as *B* is designated for testing. And we use 10-fold cross-validations to ensure that our results are reliable and report the mean performance via precision, recall, and *F*1-measure.

### 5.2. Performance Comparisons with Different Retweeting Sentiment Tendency Analyzing Methods

#### 5.2.1. Experiments with Different Feature-Based Methods

To answer the first question, we first compare the proposed framework MLNBRST with four different feature-based methods:

- pMLNBRST: considering user's profile-based features only;
- rMLNBRST: considering user's relationship-based features only;
- eMLNBRST: considering user's emotion-based features only;
- aMLNBRST: considering all features.

**Figure 2:**
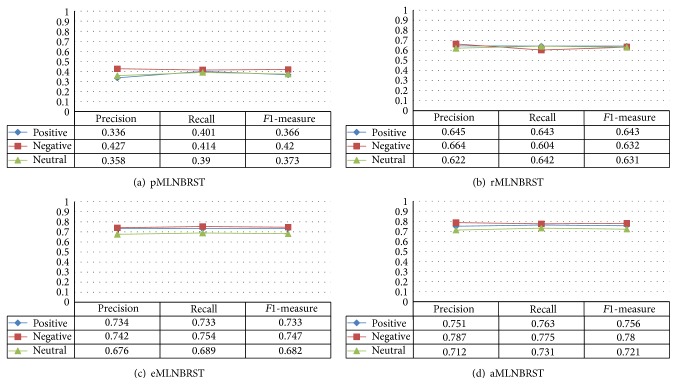
The impact of different feature sets in the proposed framework MLNBRST.

The precision, recall, and *F*1-measure of different feature-based methods are shown in Figure 2.

We draw the following observations: removing either user's relationship-based or emotion-based features may lower the model's prediction abilities obviously. Additionally, emotion-based features contribute more to analyzing user's retweeting sentiment polarity than profile- and relationship-based features since a plenty of positive or negative emotional words in retweeting contents provide a good guarantee for accuracy of emotion predicting. Besides, the system improves its performance by shifting the emphasis towards merging profile-, relationship-, and content-based features from which we found that it is difficult to predict user's retweeting emotion tendency with only its specific types of features and it is important to fuse multidimensional features reasonably.

#### 5.2.2. Experiments with Other Sentiment Tendency Analyzing Methods

**Figure 3:**
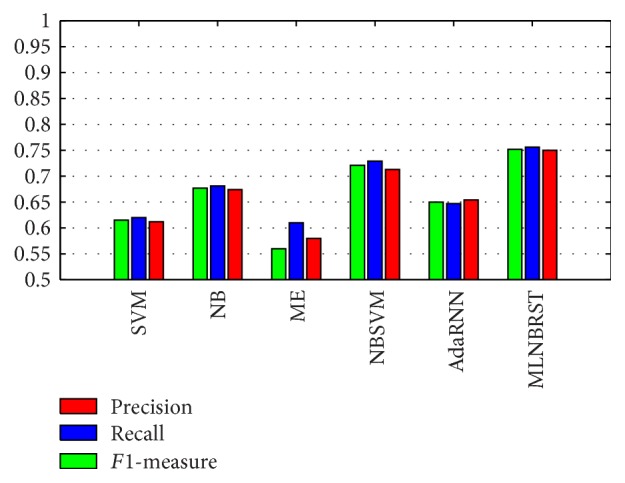
Precisions, recalls, and *F*1-measures of different methods.

Since support vector machine (denoted as SVM), Naïve Bayes (denoted as NB), and maximum entropy models (denoted as ME) are most commonly used in sentiment classification among various machine learning techniques [26], therefore, on the basis of our proposed features, we compare the proposed framework MLNBRST with SVM, NB, and ME, as well as NBSVM used in [16] which is an improved SVM classifier and Adaptive Recursive Neural Network (denoted as AdaRNN) used in [20], to answer the first question. Due to space restrictions, the mean precisions, recalls, *F*1-measures of the aforementioned studies and our best one are depicted in Figure 3.

From Figure 3, it can be found that the proposed approach shows the best results from the point of view of precision, recall, and *F*1-measure. Maximum entropy method achieves the worst results because it strongly relies on corpus. Since support vector machine is only applicable to a small-size training dataset, therefore, Naïve Bayes is more suitable for sentiment classification than support vector machine in terms of microblogging which is in line with [25]. Adaptive Recursive Neural Network method only can achieve better precision via a complete dataset. Additionally, given context of retweeting content, our proposed method stratifies different factors according to the correlations between them via a multilayer Naïve Bayes model; consequently, it performs better than Naïve Bayes and NBSVM method. Moreover, we can obtain a significant improvement on performance (+10.7% in terms of precision, 22.3% in terms of recall, and +16.9% in terms of *F*1-measure) compared with [11] which leveraged a Naïve Bayes classifier to analyze user's sentiment via 95 most frequently used emoticons in Chinese tweets. Besides, we achieve better results (+5.8% in terms of precision, +12.1% in terms of recall, and +9.9% in terms of *F*1-measure) compared with [18] which leveraged SVR (support vector regression) to classify emotions in Chinese tweets on the basis of common social network characteristics and other carefully generalized linguistic patterns.

In summary, the results in Sections 5.2.1 and 5.2.2 suggest that all improvement is significant. With the help of multilayer Naïve Bayes model based on integration of multidimensional features, the proposed framework, MLNBRST, gains significant improvement over representative different feature-based methods and baseline methods, which answers the first question.

### 5.3. Analysis of Different Factors' Impacts in MLNBRST

#### 5.3.1. Impacts of Different Retweeting Sentiment Features in MLNBRST

**Figure 4:**
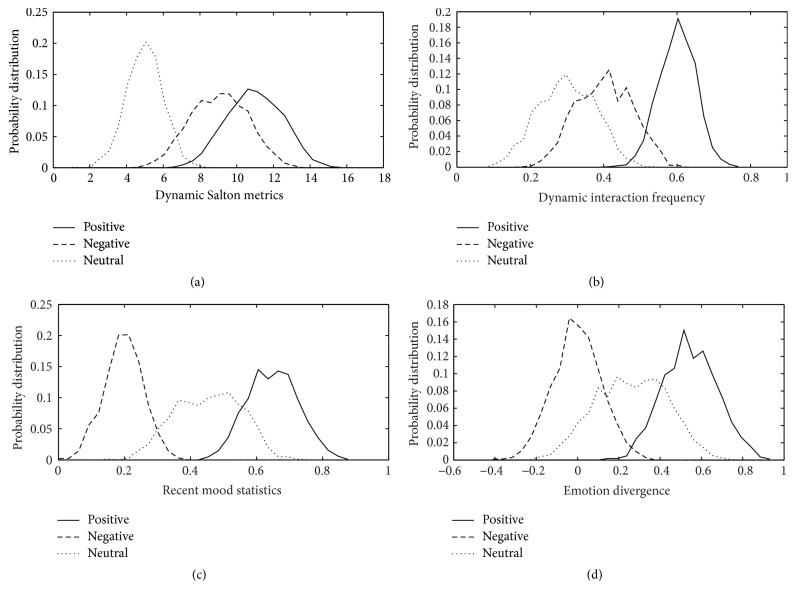
The probability distribution of different retweeting sentiment tendency features.

In this context, we first investigate the impact that the proposed retweeting sentiment features have and accordingly answer the second question. Because of the space crunch, Figures 4(a), 4(b), 4(c), and 4(d) merely illustrate the probability distribution of values of dynamic Salton metrics, dynamic interaction frequency, recent mood statistics, and emotion divergence under different retweeting sentiment tendencies (positive, negative, and neutral).

As depicted in Figure 4(a), if there is a bigger dynamic Salton metrics between two users, it is more easier to retweet casually which may lead to either positive emotion or negative emotion being included in retweeting contents. In Figure 4(b), users who interact frequently may be less possible to retweet in neutral emotion tendency with each other; instead, there may be more likely to express support or opposition to each other. And in Figure 4(c), users will have higher probability to retweet with positive emotion tendency if they are in high spirits latently, and vice versa.  Figure 4(d) shows strong evidence for the impact that emotion divergence has on user's retweeting emotion tendency. If absolute values of emotion divergence are small, the majority of users may retweet with neutral emotion. And the ratio of retweeting with negative emotion is higher if there are negative values of emotion divergence between emotion of microblogging and emotion that is expressed in user's recent states. If values of emotion divergence are positive, users are more likely to retweet with positive emotion which may be determined by users' recent mood statistics.

Furthermore, we employ information entropy theory to further explore the contributions of the proposed features to user's retweeting sentiment tendency. The information gain of the *i*th feature *f*_*i*_ is calculated as

(13) $$\begin{array}{c} \text{IG}\left(f_{i}\right)=-\underset{e\in E}{\sum }p\left(e\right)\log p\left(e\right)+\underset{v\in V_{f_{i}}}{\sum }p\left(v\right)\underset{e\in E}{\sum }p\left(e\;|\;v\right)\log p\left(e\;|\;v\right), \end{array}$$

where *e* denotes a certain emotion state which belongs to emotion set *E* = {positive, negative, neutral}, *v* denotes a value in discretized value set *V*_*f*_*i*__ of the *i*th feature *f*_*i*_, *p*(*e*) stands for the probability that emotion state *e* appears in dataset, *p*(*v*) stands for the probability that discretized value of *f*_*i*_ is equal to *v* in dataset, and *p*(*e*∣*v*) stands for the probability that emotion state *e* appears in dataset when discretized value of *f*_*i*_ is equal to *v*. Information gains of features are shown in Table 2.

As described in Table 2, information gain of user's profile-based features is lower than other relationship- and emotion-based features. Moreover, emotion-based features have higher information gains than relationship-based features. In addition, being processed based on the count of positive and negative emotional words, recent mood statistics and emotion divergence have larger information gains than positive or negative emotional words count.

From the above, it can be found that the proposed factors can be used as a good indicator of user's retweeting sentiment tendency. However, although social relationship played an important role in individual emotion which is in keeping with Fowler and Christakis's work [27], it can only distinguish between neutral and other sentiment tendencies, while emotion-based features are merely with good discriminability on positive and negative sentiment polarity. Hence, comprehensive considering on context information of retweeting content is necessary.

#### 5.3.2. Impact of Temporal Information in MLNBRST

**Figure 5:**
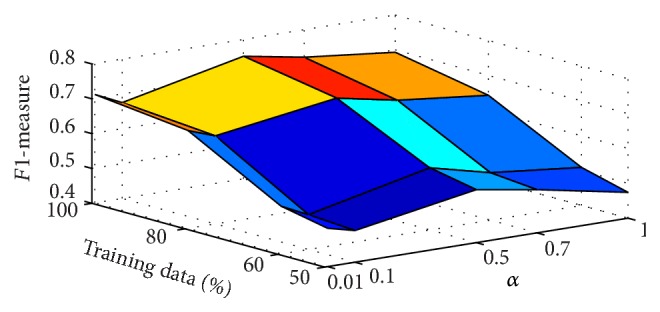
The impact of temporal information in the proposed framework MLNBRST.

To answer the second question, we also investigate how temporal information affects the performance of our method in terms of *F*1-measure by changing the time slice weight factor *α*. In this paper, *α* is varied as {0.01,0.1,0.5,0.7,1} and we carry on 10-fold cross-validations with 50%, 60%, 80%, and 100% of *A* for training so as to avoid bias brought by the sizes of the training data and the results are shown in Figure 5, where “50%,” “60%,” “80%,” and “100%” denote that we leverage 50%, 60%, 80%, and 100% of *A* for training.

It can be observed from Figure 5: when setting *α* as 1, namely, without considering temporal information, the *F*1-measure is much lower than the peak performance, and the *F*1-measure first increases greatly and then degrades rapidly after reaching a peak value with the increase *α*.

The results in Sections 5.3.1 and 5.3.2 further demonstrate the importance of proposed features and temporal information in retweeting sentiment tendency analysis, which correspondingly answers the second question.

## 6. Conclusion

In this paper, we explored the problem of finding the possible variations and analyzing user's retweeting sentiment tendency in dynamic social networks. Firstly, relationship-based features were inferred from users' dynamic Salton metrics and dynamic interaction frequency. Secondly, along with the number of positive and negative emotional words, we built recent mood statistics and emotion divergence based on time series of users' posts. And then on the basis of Naïve Bayes theory, we represented models in lower layers from profile-, relationship-, and emotion-based dimension, respectively, followed by designing a multilayer Naïve Bayes model on constructed models of different dimensions to analyze user's retweeting sentiment tendency. Finally, we ran a set of experiments on a real-world dataset to investigate the performance of our model and reported system performances in terms of precision, recall, and *F*1-measure. In general, the experimental results demonstrate the effectiveness of our proposed framework.

In future work, we will employ crowd sourcing technology to add more context information to our method to ameliorate its performance as well as increase its online application scope. Furthermore, we will speculate on what directions can be undertaken to ameliorate its performance with respect to time complexity.

## Figures and Tables

**Table 1:** Statistics of the dataset.

Features	Statistics
#users	296134
#followings	51415017
#tweets	176659
#retweets	264743

**Table 2:** Information gains of features.

Types of features	Features	Information gains
Profile-based	#bifollowers	0.254
	#followers	0.231
	#followees	0.257
	#posts	0.201
	Province	0.105
	City	0.110
	Gender	0.148
	Created time of user's account	0.092
	Verified type of user's account	0.102

Relation-based	Dynamic Salton metrics	0.485
	Dynamic interaction frequency	0.503

Emotion-based	#positive emotional words	0.647
	#negative emotional words	0.622
	Recent mood statistics	0.694
	Emotion divergence	0.723
